# Aqueous Humor Cytokine Profiling Reveals Distinct Roles for Serum Amyloid A, Interleukin-8, and Endothelin-1 in Pseudoexfoliation Syndrome and Glaucoma

**DOI:** 10.3390/ijms26041461

**Published:** 2025-02-10

**Authors:** Yoichi Kadoh, Yuji Takayanagi, Kazunobu Sugihara, Sachiko Kaidzu, Yasuyuki Takai, Masaki Tanito

**Affiliations:** Department of Ophthalmology, Shimane University Faculty of Medicine, Izumo 693-8501, Japan

**Keywords:** pseudoexfoliation syndrome, glaucoma, exfoliation glaucoma, inflammatory cytokines, aqueous humor, Serum Amyloid A (SAA), Interleukin-8 (IL-8), Endothelin-1 (ET-1), Vascular Endothelial Growth Factor (VEGF)

## Abstract

Pseudoexfoliation syndrome (PE), which is often unilateral in 60% of cases, is a risk for exfoliation glaucoma (EXG) with elevated inflammatory cytokines. This study aimed to clarify the dynamics of these cytokines in unilateral PE (u-PE) patients. This study included 20 eyes from 10 u-PE patients (PE+ eyes and fellow PE− eyes) and 20 eyes from 10 cataract patients without PE (control group). Clinical parameters, including age, visual acuity, and intraocular pressure, were assessed. Anterior chamber aqueous humor cytokine levels (IL-8, SAA, ET-1, VEGF) were measured and compared among groups. SAA was elevated in PE+ eyes compared to PE− and control eyes. IL-8 and ET-1 were elevated in both PE+ and PE− eyes compared to controls. IL-8 was associated with worsening visual acuity, while ET-1 correlated inversely. Our findings suggest that SAA is associated with the manifest disease while IL-8 and ET-1 could be early biomarkers for PE and therapeutic targets to prevent glaucomatous damage, as these markers appear in the aqueous humor even before pseudoexfoliation material becomes clinically evident. These results may enable earlier diagnosis and therapeutic intervention before the clinical onset of PE in patients with risk factors.

## 1. Introduction

Glaucoma is a neurodegenerative disorder characterized by irreversible damage to the optic nerve and is one of the leading causes of blindness and low vision. The global prevalence of the disease among individuals aged 40 and older is approximately 3.5% [1]. In the early stages of glaucoma, patients are often asymptomatic, which complicates timely management. EXG, the most prevalent type of secondary glaucoma, progresses more rapidly than primary open-angle glaucoma (POAG) and responds less effectively to pharmacological treatment or surgery, increasing the risk of blindness [2,3]. Early diagnosis and treatment are therefore essential.

Pseudoexfoliation syndrome (PE) is a systemic age-related disorder characterized by the abnormal accumulation of extracellular fibrillar material in various ocular and systemic tissues [4,5,6]. Although often asymptomatic, PE frequently develops elevated intraocular pressure (IOP) during its course and progresses to a condition known as EXG [7,8]. In long-term observational studies, it has been reported that 44% of patients with PE progress to glaucoma or ocular hypertension within 15 years [9]. Notably, PE frequently presents unilaterally, with one eye demonstrating classic features of the disease while the fellow eye remains clinically unaffected, providing a unique opportunity to investigate localized ocular pathophysiology [4]. In 81% of patients with clinically u-PE, pseudoexfoliation material has been detected ultrastructurally in the supposedly unaffected eye [10].

Cytokines are small, cell-signaling proteins that play crucial roles in mediating inflammation, tissue remodeling, and vascular regulation in various diseases, including ocular conditions such as glaucoma [11] and uveitis [12]. Glaucoma is increasingly recognized as a condition influenced by inflammatory processes. Pro-inflammatory cytokines contribute to tissue remodeling and inflammatory microenvironments in glaucomatous eyes [13]. Specifically, Interleukin-8 (IL-8) levels are linked to immune activation and optic nerve damage, underscoring inflammation as a key player in glaucoma pathogenesis [14]. We previously reported that cytokines such as Transforming Growth Factor (TGF)-β1 and IL-8 were significantly elevated in the aqueous humor of patients with POAG and EXG, correlating with intraocular pressure (IOP) and disease progression [15]. Furthermore, systemic redox status and ocular oxidative stress are significantly associated with POAG and EXG [16]. Analyzing the cytokine profile in the aqueous humor of PE patients may offer valuable insights into the disease’s underlying mechanisms. Previous studies have highlighted the involvement of specific cytokines in the pathogenesis of PE and EXG, but the precise role of these molecules remains unclear.

Serum Amyloid A (SAA) is a pro-inflammatory acute-phase protein implicated in oxidative stress and amyloid deposition, which are both thought to contribute to the progression of PE [17]. The concentration of SAA in the anterior chamber has been elucidated in several previous studies [15,18,19]. Human SAA has four subtypes: SAA1, SAA2, SAA3, and SAA4. SAA1 and SAA2 are acute-phase proteins that increase during inflammation (i.e., acute-phase SAA) and contribute to AA amyloidosis. SAA3 is non-functional in humans (a pseudogene). SAA4 is constitutively expressed rather than being an acute-phase protein (i.e., constitutive SAA), but its function remains incompletely understood. Increased expression of SAA1/2 has been observed in the trabecular meshwork tissue and cells of glaucoma patients, and it has been shown that this increase contributes to the glaucoma phenotype characterized by elevated intraocular pressure [20]. Notably, SSA4 is known as a common marker of persistent inflammation in neovascular age-related macular degeneration (nAMD) and polypoidal choroidal vasculopathy (PCV) [21]. Proteomic analysis of aqueous humor in smokers revealed an increased expression of inflammatory proteins, including SAA1/2, suggesting their potential as biomarkers and therapeutic targets for smoking-related ocular diseases [22]. IL-8, a potent neutrophil chemoattractant, has been associated with inflammatory responses and may contribute to the inflammatory milieu observed in PE [23]. Endothelin-1 (ET-1), a vasoconstrictive peptide, has been shown to impair ocular blood flow and exacerbate glaucomatous optic neuropathy [24]. Vascular Endothelial Growth Factor (VEGF), a key mediator of angiogenesis, is also elevated in several ocular diseases and may play a role in the vascular dysregulation seen in PE [25].

Despite these findings, comparative analyses of aqueous humor cytokine profiles in u-PE remain limited. This study aims to elucidate the role of SAA, IL-8, ET-1, and VEGF in the pathophysiology of PE by analyzing their concentrations in the aqueous humor of affected and unaffected eyes. By identifying potential molecular markers or therapeutic targets, this research seeks to enhance our understanding of PE and contribute to the development of targeted treatments.

## 2. Results

The demographic data for the four groups [PE+ (pseudoexfoliation positive eye), PE− (pseudoexfoliation negative eye), CT-R (control, right eye), and CT-L (control, left eye)] are presented in Table 1. The study included 20 eyes of 10 participants in each unilateral PE and control group, therefore each of the four groups included 10 eyes. The mean age at the aqueous humor sampling was similar across groups, with no significant difference (PE+: 78.7 ± 6.1, PE−: 78.8 ± 6.0, CT-R: 78.7 ± 4.9, CT-L: 78.7 ± 4.9; *p* > 0.99). The proportion of male participants was 30% (3/10) in each group, with no significant difference (*p* = 1.00). A significant difference in laterality (right versus left eyes) was observed among the groups (*p* < 0.0001). All participants in the CT-R group had their right eyes examined, while all participants in the CT-L group had their left eyes examined. The PE+ and PE− groups had an equal distribution of right and left eyes (50% each). Visual acuity (VA), measured in logMAR, did not differ significantly between the groups (*p* = 0.82). The mean VA values were 0.27 ± 0.25 for the PE+ group, 0.23 ± 0.15 for the PE− group, 0.39 ± 0.49 for the CT-R group, and 0.38 ± 0.31 for the CT-L group. IOP was also comparable among the groups (*p* = 0.30). The mean IOP values were 16.3 ± 4.3 mmHg for the PE+ group, 15.7 ± 4.9 mmHg for the PE− group, 13.8 ± 1.5 mmHg for the CT-R group, and 14.1 ± 1.8 mmHg for the CT-L group. A statistically significant difference was observed in the mean number of medications used between the groups (*p* = 0.0139), with the PE+ group exhibiting a higher mean number of medications compared to the PE− group.

The levels of cytokines SAA, IL-8, ET-1, and VEGF in the aqueous humor were compared between the PE+ and PE− groups, as summarized in Table 2. A significant difference in SAA levels was observed between the groups, with the PE+ group showing higher levels (2.54 ± 3.15 µg/mL) compared to the PE− group (0.39 ± 0.73 µg/mL, *p* = 0.00442). No significant differences were observed in the levels of IL-8, ET-1, or VEGF between the two groups. The levels of SAA, IL-8, ET-1, and VEGF were compared between the CT-R and CT-L groups (Table 3). No significant differences were observed in any of the biomarkers between the two groups, including SAA (*p* = 0.44), IL-8 (*p* = 0.12), ET-1 (*p* = 0.50), and VEGF (*p* = 0.07).

The levels of biomarkers SAA, IL-8, ET-1, and VEGF were compared across the three groups: PE+, PE−, and control (Table 4). For SAA, the difference among the groups was not statistically significant (*p* = 0.07). IL-8 levels showed a significant difference among the groups (*p* = 0.0049). Pairwise comparisons indicated that both the PE+ group (*p* = 0.0126) and the PE− group (*p* = 0.0435) had significantly higher IL-8 levels compared to the CT group. No significant difference was observed between the PE+ and PE− groups (*p* = 0.96). ET-1 levels also differed significantly among the groups (*p* = 0.0052). The PE+ group exhibited significantly higher ET-1 levels compared to the control group (*p* = 0.0073). No significant differences were observed between the PE+ and PE− groups (*p* = 0.47) or between the PE− and CT groups (*p* = 0.1347). For VEGF, no significant differences were observed among the groups (*p* = 0.51).

**Figure 1:**
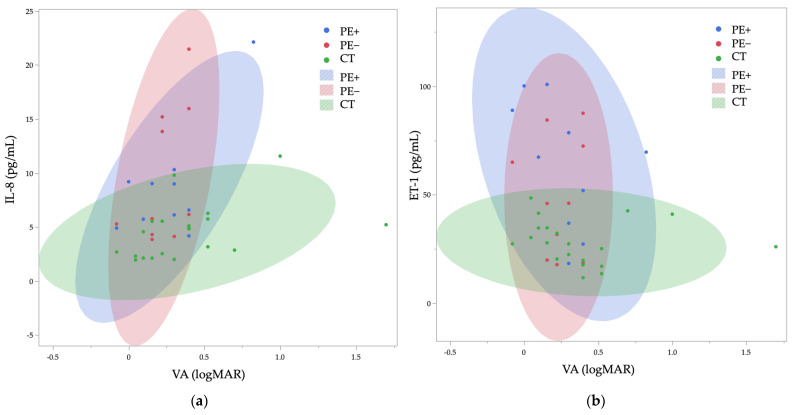
Relationship between VA and IL-8 (**a**), ET-1 (**b**). The shaded area represents the 95% probability ellipse.

Table 5 summarizes the correlation analysis between SAA, IL-8, ET-1, and VEGF levels. No significant correlations were observed among the cytokines. The correlation analysis between clinical parameters (age, VA, IOP) and cytokines (SAA, IL-8, ET-1, and VEGF) revealed significant associations between VA and IL-8 (ρ = 0.24, *p* = 0.046) (Table 6). A significant correlation was found between VA and IL-8 (ρ = 0.24, *p* = 0.046). Additionally, a significant correlation was observed between VA and ET-1 (ρ = −0.22, *p* = 0.026). No significant correlations were identified between other biomarkers (SAA, VEGF) and clinical parameters. The relationship between VA and IL-8, as well as ET-1, is shown in Figure 1a,b. It can be observed that the association between cytokines and VA differs among the PE+, PE−, and CT groups.

## 3. Discussion

This study provides valuable insights into the relationships between clinical parameters and cytokine levels in aqueous humors from u-PE patients. SAA levels were significantly elevated in eyes with PE+ compared to fellow PE− eyes (Table 2). The IL-8 levels were higher in both the PE+ eyes and PE− eyes compared to the control group. ET-1 levels were elevated in the PE+ eyes, with no significant difference observed in the PE− eyes. There were no significant differences in the levels of IL-8 and ET-1 between the PE+ eyes and their corresponding fellow PE− eyes (Table 4). IL-8 levels were positively correlated with worsening VA, while ET-1 levels were negatively correlated with VA (Table 6).

The SAA levels in the eyes with PE (2.54 μg/mL) were significantly higher than those in their corresponding fellow eyes (0.39 μg/mL) (Table 3). In previous studies, aqueous humor levels of SAA were reported to be 5–10 μg/mL in juvenile idiopathic arthritis-associated anterior uveitis [19]. Thus, although the SAA level in PE+ eyes is not as high as in acute uveitis, it is considered quite elevated for a chronic disease. SAA is an acute-phase reactant that modulates inflammatory pathways and immune cell recruitment. Its role in IOP elevation may involve the induction of fibrosis in the trabecular meshwork, pro-inflammatory effects, and oxidative stress. Inflammatory mediators, including SAA, have been reported to be overexpressed in glaucomatous eyes, correlating with reduced aqueous outflow [11]. Elevated SAA levels are also associated with inflammatory conditions linked with secondary ocular hypertension [15]. SAA has been implicated in oxidative stress mechanisms that damage ocular tissues, particularly in the trabecular meshwork. Accordingly, SAA appears to contribute to IOP elevation in EXG through its roles in inflammation, fibrosis, and oxidative stress.

IL-8 levels were elevated in both PE+ eyes and PE− eyes compared to the control group. ET-1 levels were higher in PE+ eyes but showed no significant differences in PE− eyes (Table 4). IL-8 is a pro-inflammatory cytokine that promotes neutrophil migration and activation. Elevation of IL-8 in both PE+ and PE− eyes in this study suggests the presence of chronic inflammation in the eye, regardless of the presence of clinically detectable pseudoexfoliation material. The significantly higher IL-8 levels in the PE+ group suggest that pseudoexfoliation material may induce stress on corneal endothelial cells and retinal ganglion cells, triggering inflammatory pathways. This inflammation likely contributes to Schlemm’s canal obstruction and elevated IOP, playing a critical role in the pathogenesis of EXG. Elevation of IL-8 may also be associated with the loss of Schlemm’s canal endothelial cells, a hallmark of EXG rather than the POAG [26]. ET-1 is a potent vasoconstrictor peptide implicated in optic nerve blood flow dysregulation and neurodegeneration. ET-1, also known to be a strong constrictor of TM cells [27], was significantly elevated in the PE+ group compared to the control group. However, no significant differences in ET-1 levels were observed in the PE− group. This finding suggests that pseudoexfoliation material may further promote ET-1 production, emphasizing the role of vascular constriction and ischemic stress in EXG progression.

PE is clinically classified based on anterior lens capsule findings. A definitive diagnosis is possible only in late-stage PE, marked by focal defects in precursor material, typically in the upper nasal quadrant. Early signs that may suggest PE include iris pigment loss, transillumination defects, melanin dispersion in the anterior chamber, deposition on anterior structures, insufficient mydriasis, and asymmetric manifestation without other causes [4]. In our results, the PE+ eyes showed significantly higher SAA levels compared to their corresponding fellow eyes, while IL-8 and ET-1 levels were elevated in both the affected and fellow eyes compared to the control group. These findings suggest that the increase in SAA levels does not occur until the development of the classic PE state, characterized by the deposition of PE fibers. In PE, despite the characteristic accumulation of fibrillar deposits, direct colocalization with SAA has not been conclusively demonstrated. However, given its function in inflammation, SAA may be indirectly linked to the extracellular matrix abnormalities observed in PE. Furthermore, elevated SAA levels—systemically in PE patients with overt inflammatory manifestations and locally in the aqueous humor of uveitis patients—highlight its potential as a biomarker for ocular inflammation. Further studies are needed to elucidate the mechanistic pathways and assess therapeutic implications. In contrast, the elevation of IL-8 and ET-1 levels appears to occur from the early preclinical stages of PE, during which microfibril production and melanocyte granule proliferation begin. The findings indicate that IL-8 and ET-1 may serve as important biomarkers in EXG pathophysiology. Targeting IL-8 and ET-1 could potentially lead to the development of novel therapeutic strategies. Systemic diseases such as diabetes, rheumatoid arthritis, and cardiovascular disease are suggested to influence local inflammatory conditions and cytokine levels, including IL-8 and SAA [28,29]. Therefore, the association between aqueous humor cytokines and PE observed in this study may be influenced by systemic PE deposition-related inflammation affecting other organs.

A positive correlation was observed between worsening VA and IL-8 levels, while an inverse correlation was observed between VA and ET-1 levels (Table 6). Because IL-8 was also elevated in the fellow eyes of patients with PE, it is possible that IL-8 levels increase before the clinical manifestation of PE and contribute to worsening VA [15,23,30]. IL-8 is a critical molecule in the pathogenesis and progression of PE and EXG. Its excessive expression has been shown to trigger inflammatory responses and abnormal extracellular matrix formation. These processes may potentially lead to optic nerve damage and vision loss. In contrast, ET-1 plays a significant role in the pathophysiology of PE and EXG [31,32]. Notably, although elevated levels of ET-1 may be associated with the progression of PE and EXG, they may not directly affect central vision despite the presence of visual field deterioration.

This study has several limitations. First, the sample size of 20 eyes per group is relatively small, which may limit the generalizability of our findings. The SAA concentration is measured as a whole, treating all SAA subtypes as identical, and subtype-specific analysis has not been conducted. The statistical power for the comparison of SAA between the PE+ and PE− groups is approximately 0.5. Therefore, the lack of significant differences in this study should be interpreted with consideration of the potential impact of low statistical power. All participants were of Japanese ethnicity, and thus, our results may not be applicable to other populations. Another limitation is that other factors that may influence aqueous humor cytokine levels, such as the effects of smoking [22], were not examined. The inconsistency in the timing of aqueous humor sampling could be a weak point of this study, considering the possible diurnal variations in cytokine levels. Finally, we measured only a limited set of cytokines, and further studies should examine a wider range of inflammatory mediators.

## 4. Materials and Methods

### 4.1. Subjects

The current study procedures were carried out in accordance with the Declaration of Helsinki. The institutional review board of Shimane University Hospital approved the research (No. 20131216-1, approved on 27 January 2014). All participants provided written informed consent for inclusion in the study. Aqueous humor samples were obtained from 40 eyes of 20 Japanese subjects (10 eyes with PE+ and 10 eyes with PE− from 10 unilateral PE patients) and 20 eyes (10 eyes with CT-R and 10 eyes with CT-L) from 10 non-glaucomatous control. All subjects underwent ophthalmologic examinations including measurements of best-corrected visual acuity (VA), IOP measured by Goldmann applanation tonometry, and slit-lamp, gonioscopic, and funduscopic examinations under pupillary dilation. PE+ was defined by an open iridocorneal angle, and the presence of characteristic pseudoexfoliation material deposits on the anterior capsule and/or pupillary margin only in one eye. PE− was defined, as was the contralateral eye of a PE+ eye with an open iridocorneal angle, and no deposition of pseudoexfoliation material was observed under careful slit-lamp examination. The control eye was defined by the absence of glaucomatous optic neuropathy, no history of IOP exceeding 21 mm Hg, and the presence of cataracts that required cataract surgery in both eyes. Except for cataracts and/or glaucoma, no subjects had ocular pathologies such as clinically detectable ocular inflammation, infection, neuropathies, retinopathies, or maculopathies. The demographic data of the subjects, including age, sex, IOP measured on the day before the surgery, and number of glaucoma medication use are recorded.

### 4.2. Measurement of Cytokine Levels

The collection of aqueous humor samples and cytokine measurement were described previously [15,16]. Aqueous humor samples (100 to 200 μL) were aspirated at the beginning of glaucoma or cataract surgery through a limbal paracentesis using a 0.5-mL syringe with a 30-gauge needle (BD Japan, Tokyo, Japan), with care taken to prevent blood and intraocular tissue contamination. After obtaining the aqueous humor samples, the anterior chamber was reformed with a balanced salt solution and the planned surgeries were performed. All samples were immediately frozen and stored at −80 °C until the analyses were performed. Cytokine concentrations were analyzed using a multiplex bead immunoassay system (Procarta Cytokine Assay Kit; Affymetrix, Inc., Santa Clara, CA, USA), based on multiplexing technology (xMAP; Luminex, Austin, TX, USA), according to the manufacturer’s instructions. The data were acquired using a Luminex-compatible workstation and its manager software (Bio-Plex workstation and version 6.0 software; Bio-Rad, Tokyo, Japan), according to the manufacturer’s instructions. To analyze four cytokines related to the inflammatory process and vascular function (IL-8, SAA, ET-1, and VEGF-A), 25 µL of undiluted aqueous humor samples were analyzed simultaneously. Based on the information provided by the manufacturer, the multiplex assay kit can quantitatively measure multiple cytokines from as little as 25 µL of bodily fluids, with a lower limit of detection of 1 pg/mL per cytokine. Each sample was run as a single measurement for the limited quantity of collected aqueous humor.

### 4.3. Statistical Analysis

For comparisons between PE+ and PE−, and between CT-R and CT-L, the differences in continuous data, i.e., age, VA, IOP, and the levels of IL-8, SAA, ET-1, and VEGF, were calculated using the Wilcoxon’s signed rank test. For comparisons between PE+, PE−, and CT (i.e., CT-R + CT-L), the differences in continuous data were calculated using Kruskal–Wallis test and the difference in the categorical datum, i.e., sex, was calculated using the G test. *p* values of 0.05 were considered statistically significant. The correlations between clinical backgrounds and cytokine levels were assessed using Spearman’s correlation test. We performed all statistical analyses using JMP Pro statistical software version 17.2 (SAS Institute, Inc., Cary, NC, USA). All reported *p* values are two-sided. The data are expressed as the means ± standard deviation (SD) for continuous variables and in numbers and percentages for categorical variables. For the statistical analyses, the decimal VA was converted to the logarithm of the minimum angle of resolution (logMAR).

## 5. Conclusions

In conclusion, our findings demonstrate that SAA, IL-8, and ET-1 have distinct roles in PE and its progression to EXG. SAA appears linked to manifest PE and IOP elevation, while IL-8 and ET-1, elevated even in fellow eyes, suggest their roles in early inflammation and vascular changes. These cytokines could serve as biomarkers for preclinical PE and targets for preventing EXG progression. Particularly, given the correlation between IL-8, ET-1, and visual acuity, these findings suggest that monitoring these cytokines could be clinically useful for at-risk identification and personalized therapy for PE/EXG, aiming to preserve visual function.

## Figures and Tables

**Table 1:** Demographic and Clinical Characteristics of Study Participants.

Parameters	PE+	PE−	CT-R	CT-L	*p*-Value
N	10	10	10	10	-
Age (years)	78.7 ± 6.1	78.8 ± 6.0	78.7 ± 4.9	78.7 ± 4.9	>0.99
Mean ± SD	78.7 ± 6.1	78.8 ± 6.0	78.7 ± 4.9	78.7 ± 4.9	>0.99
Men, n (%)	3 (30.0%)	3 (30.0%)	3 (30.0%)	3 (30.0%)	1.00
Right eye, n (%)	5 (50.0%)	5 (50.0%)	10 (100.0%)	0 (0.0%)	<0.0001 **
VA (logMAR)	0.27 ± 0.25	0.23 ± 0.15	0.39 ± 0.49	0.38 ± 0.31	0.82
IOP (mmHg)	16.3 ± 4.3	15.7 ± 4.9	13.8 ± 1.5	14.1 ± 1.8	0.30
Medication number	2.9 ± 0.4	1.4 ± 0.4	-	-	0.0139 *

Values are expressed in mean ± standard deviation or n (%). *p* values are calculated by the Kruskal–Wallis test for continuous variables and the chi-square test for categorical variables. The * and ** indicate *p* < 0.05 and *p* < 0.01, respectively.

**Table 2:** Aqueous Humor Cytokine Levels in PE+ and PE− Eyes.

Parameters	PE+	PE−	*p*-Value
SAA (μg/mL)	2.54 ± 3.15	0.39 ± 0.73	0.044 *
IL-8 (pg/mL)	8.71 ± 5.14	9.59 ± 6.38	0.40
ET-1 (pg/mL)	64.0 ± 29.5	48.9 ± 27.1	0.23
VEGF (pg/mL)	255.6 ± 112.4	253.4 ± 62.3	0.51

Values are expressed in mean ± standard deviation. *p* values are calculated by Wilcoxon’s signed rank test. The * indicates *p* < 0.05.

**Table 3:** Comparison of Cytokine levels Between the Control Right and Left Eyes.

Parameters	CT-R	CT-L	*p*-Value
SAA (μg/mL)	0.53 ± 1.00	0.60 ± 1.22	0.44
IL-8 (pg/mL)	4.18 ± 3.08	4.90 ± 2.07	0.12
ET-1 (pg/mL)	29.8 ± 12.1	26.4 ± 8.0	0.50
VEGF (pg/mL)	206.5 ± 74.7	256.0 ± 93.5	0.07

Values are expressed in mean ± standard deviation. *p* values are calculated by Wilcoxon’s signed rank test.

**Table 4:** Aqueous Humor Cytokine Levels Across Study Groups.

Parameters	PE+	PE−	CT	*p*-Value
SAA (μg/mL)	2.54 ± 3.15	0.39 ± 0.73	0.57 ± 1.09	0.07
IL-8 (pg/mL)	8.71 ± 5.14	9.59 ± 6.38	4.54 ± 2.58	0.0049 **
		vs. PE+ *p* = 0.96	vs. PE+ *p* = 0.0126 *	
			vs. PE− *p* = 0.0435 *	
ET-1 (pg/mL)	64.0 ± 29.5	48.9 ± 27.1	28.0 ± 10.2	0.0052 **
		vs. PE+ *p* = 0.47	vs. PE+ *p* = 0.0073 **	
			vs. PE− *p* = 0.1347	
VEGF (pg/mL)	255.6 ± 112.4	253.4 ± 62.3	231.3 ± 86.2	0.51

Values are expressed in mean ± standard deviation. *p* values are calculated by the Kruskal–Wallis test. *p* values for pairwise comparisons are calculated by the Steel–Dwass test. The * and ** indicate *p* < 0.05 and *p* < 0.01, respectively.

**Table 5:** Correlation Analysis of SAA, IL-8, ET-1, and VEGF Levels.

*p*\ρ	SAA	IL-8	ET-1	VEGF
SAA		0.31	0.22	−0.04
IL-8	0.12		0.38	0.22
ET-1	0.62	0.14		0.04
VEGF	0.54	0.12	0.88	

*p* and ρ values are calculated by Spearman’s correlation coefficient test.

**Table 6:** Correlation Between Clinical Parameters and Cytokines.

Parameters	Age (/y)		VA (/logMAR)		IOP (/mmHg)	
*p*/ρ	ρ	*p*	ρ	*p*	ρ	*p*
SAA	−0.05	0.29	0.15	0.21	0.09	0.99
IL-8	0.14	0.43	0.24	0.046 *	0.15	0.21
ET-1	0.12	0.94	−0.22	0.026 *	0.18	0.10
VEGF	0.26	0.06	−0.02	0.76	0.03	0.33

*p* and ρ values are calculated by Spearman’s correlation coefficient test. The * indicates *p* < 0.05.

## Data Availability

Data are fully available upon reasonable request to the corresponding author.
